# Religious Service Attendance and Mortality among Adults in the United States with Chronic Kidney Disease

**DOI:** 10.3390/ijerph182413179

**Published:** 2021-12-14

**Authors:** Marino A. Bruce, Roland J. Thorpe, Dulcie Kermah, Jenny Shen, Susanne B. Nicholas, Bettina M. Beech, Delphine S. Tuot, Elaine Ku, Amy D. Waterman, Kenrik Duru, Arleen Brown, Keith C. Norris

**Affiliations:** 1 Program for Research on Faith, Justice and Health, Department of Behavioral and Social Sciences, College of Medicine, University of Houston, Houston, TX 77204, USA; rthorpe@jhu.edu (R.J.T.J.); bmbeech@central.uh.edu (B.M.B.); kcnorris@mednet.ucla.edu (K.C.N.); 2 Department of Health Systems and Population Health Sciences, College of Medicine, University of Houston, Houston, TX 77204, USA; 3 University of Houston Population Health, Houston, TX 77204, USA; 4 Program for Research on Men’s Health, Hopkins Center for Health Disparities Solutions, Johns Hopkins Bloomberg School of Public Health, Baltimore, MD 21205, USA; 5 Charles R. Drew University of Medicine and Science, Los Angeles, CA 90059, USA; dulciekermah@cdrewu.edu; 6 Department of Medicine, David Geffen School of Medicine, Los Angeles, CA 90095, USA; jshen@lundquist.org (J.S.); SuNicholas@mednet.ucla.edu (S.B.N.); kduru@mednet.ucla.edu (K.D.); abrown@mednet.ucla.edu (A.B.); 7 The Lundquist Institute, Harbor-UCLA Medical Center, Torrance, CA 90509, USA; 8 Division of Nephrology, University of California, San Francisco, CA 94143, USA; Delphine.tuot@ucsf.edu (D.S.T.); elaine.ku@ucsf.edu (E.K.); 9 Center for Vulnerable Populations, Priscilla Chan and Mark Zuckerberg San Francisco General Hospital, University of California, San Francisco, CA 94110, USA; 10 Kidney Health Research Institute, University of California, San Francisco, CA 94143, USA; 11 Department of Surgery, Houston Methodist Hospital, Houston, TX 77030, USA; awaterman@houstonmethodist.org

**Keywords:** religiosity, CKD, mortality, NHANES, population health

## Abstract

Religion and related institutions have resources to help individuals cope with chronic conditions, such as chronic kidney disease (CKD). The purpose of this investigation is to examine the association between religious service attendance and mortality for adults with CKD. Data were drawn from NHANES III linked to the 2015 public use Mortality File to analyze a sample of adults (*n* = 3558) who had CKD as defined by a single value of estimated glomerular filtration rate (eGFR) < 60 mL/min/1.73 m^2^ using the Chronic Kidney Disease Epidemiology Collaboration (CKD-EPI) equation and/or albumin-to-creatinine ratio ≥17 mg/g for males or ≥25 for females. All-cause mortality was the primary outcome and religious service attendance was the primary independent variable. Cox proportional hazards models were estimated to determine the association between religious service attendance and mortality. The mortality risks for participants who attended a service at least once per week were 21% lower than their peers with CKD who did not attend a religious service at all (HR 0.79; CI 0.64–0.98). The association between religious service attendance and mortality in adults with CKD suggest that prospective studies are needed to examine the influence of faith-related behaviors on clinical outcomes in patients with CKD.

## 1. Introduction

Chronic kidney disease (CKD) is a progressive and frequently irreversible health condition that adversely impacts the quality of life for patients and their families worldwide [1]. The prevalence of CKD has stabilized in recent years; however more than one in seven adults in the United States (US) have CKD [2,3,4]. The burden of CKD has multiple dimensions and individuals living with this condition can experience stress from numerous sources. Complex disease management protocols that include multiple medications and uncomfortable food and fluid restrictions can be burdensome and the potential economic and societal costs associated with this condition are considerable, as compromised kidney function is associated with reduced productivity and life expectancy [1,5,6]. The stress experienced by individuals with CKD can be significant and associated with declines in physiological and psychological functioning [7,8,9].

**Figure 1:**
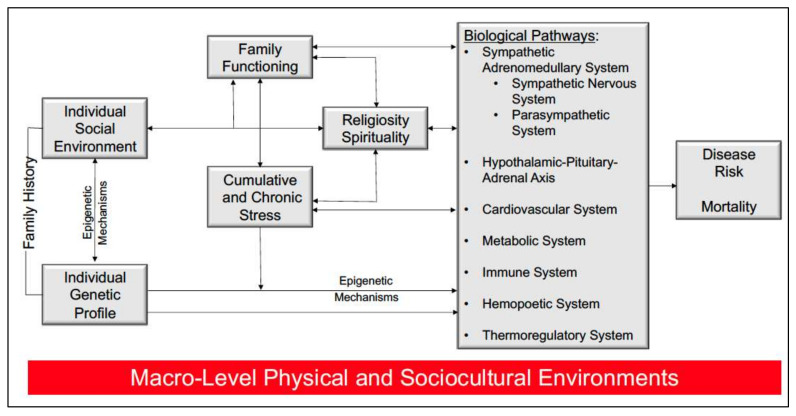
Biopsychosocial model of disease risk and mortality that emerged from Bruce and Thorpe [24].

**Figure 2:**
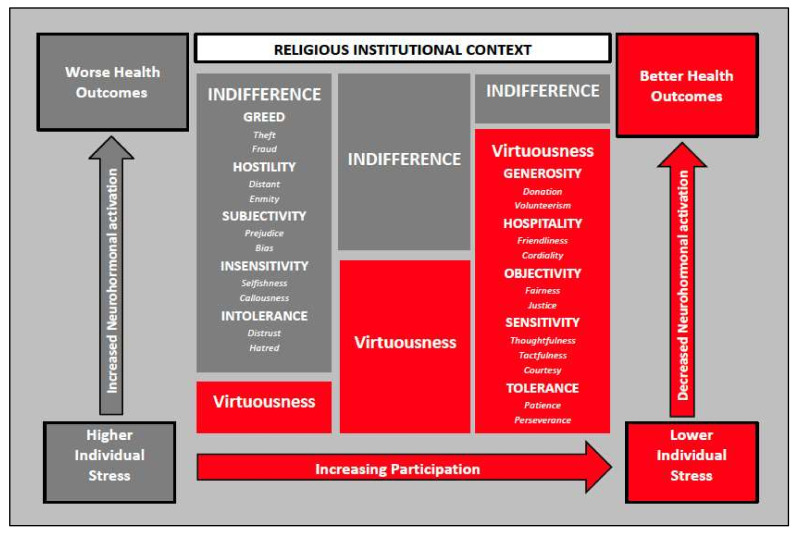
Religious institutional context and health outcomes adapted from Bruce and colleagues [34].

Religion and related institutions can provide psychological, emotional, and social resources to help individuals and families cope with chronic diseases. A growing number of studies [10,11,12,13,14,15] in the United States [11,15], Brazil [12,14], and other countries [10,13] have begun to explore religiosity or spirituality and its respective impact on outcomes in patients with advanced CKD on dialysis. Religiosity involves behaviors associated with the norms, traditions, and doctrines of an organized religion, whereas spirituality refers to the within-person experiences with, awareness of, and connection to a greater power or force [16,17]. Religious institutions (i.e., churches, mosques, and temples) are settings where individuals can participate in religious traditions and have spiritual experiences, as well as receive social, emotional, and economic support [18,19,20]. Religious service attendance has been associated with mortality in the general population [21,22,23]; however, the impact of attending religious services on clinical outcomes among individuals with chronic health conditions is less clear. The biopsychosocial model depicted in Figure 1 emerges from Bruce and Thorpe [24], and integrates concepts from Seeman and Crimmins [25], Bruce, Griffith and Thorpe [26], and Saban and colleagues [27] to specify potential pathways through which religiosity and spirituality can have implications for physiologic functioning, disease risk, and mortality. It has been suggested that religious institutions and services provide support and affirmative messages useful for coping during stressful circumstances [20,24,28,29,30]. Pargament and colleagues note diseases such as CKD can be associated with positive or negative forms of religious coping [31] that can be informed by religious service attendance. Perceptions or beliefs about the presence (positive) or absence (negative coping) of a higher power has been found to be related to health behaviors [32] and quality of life [12,33], which are often linked to disease progression and complications. Bruce and colleagues [34] suggest the pursuit of peace, harmony and balance though contemplative practices can be useful in the treatment of mental and physical conditions as they can retard disease progression and potentially prolong longevity. Religious organizations and services often encourage contemplative practices (i.e., prayer and meditation) and adoption of virtuous living that can reduce the adverse effects of stress, and our study is guided by a conceptual framework adapted from Bruce et al. [34] (see Figure 2).

To our knowledge, there have not been studies conducted to examine the role of religiosity or spirituality on outcomes for patients with CKD who are not on dialysis. It has been suggested that the traditional cardiovascular risk factors for the general population may differ in patients with different stages of CKD with higher levels of blood pressure, lipid levels, and/or body mass index being associated with increased longevity in persons with advanced stages of CKD [35,36,37]. Given the altered epidemiology of cardiovascular risk factors and mortality in patients with CKD, it is not clear what role religious service attendance may play regarding mortality in patients with CKD. The purpose of this study is to examine the association between religious service attendance and all-cause mortality for individuals with CKD in a representative sample of adults in the US. We hypothesized that increased religious service attendance is associated with a lower mortality rate among participants with CKD.

## 2. Material and Methods

### 2.1. Data

Data for our analysis were drawn from the Third National Health and Nutrition Examination Survey, 1988–1994 (NHANES III), a representative sample of civilian, non-institutionalized persons in the US from 89 random locations [38]. Respondents in the NHANES III were selected and recruited using a stratified, multistage probability sampling design. The NHANES III was selected since it would provide long-term participant follow-up allowing for a robust assessment of mortality. Data from participants were collected via in-person home interviews in which sample members provided information about their health history, health behaviors, risk factors, and health conditions. NHANES III sample members also provided biological and physiological data during a detailed physical examination at a mobile examination center [38,39,40].

**Figure 3:**
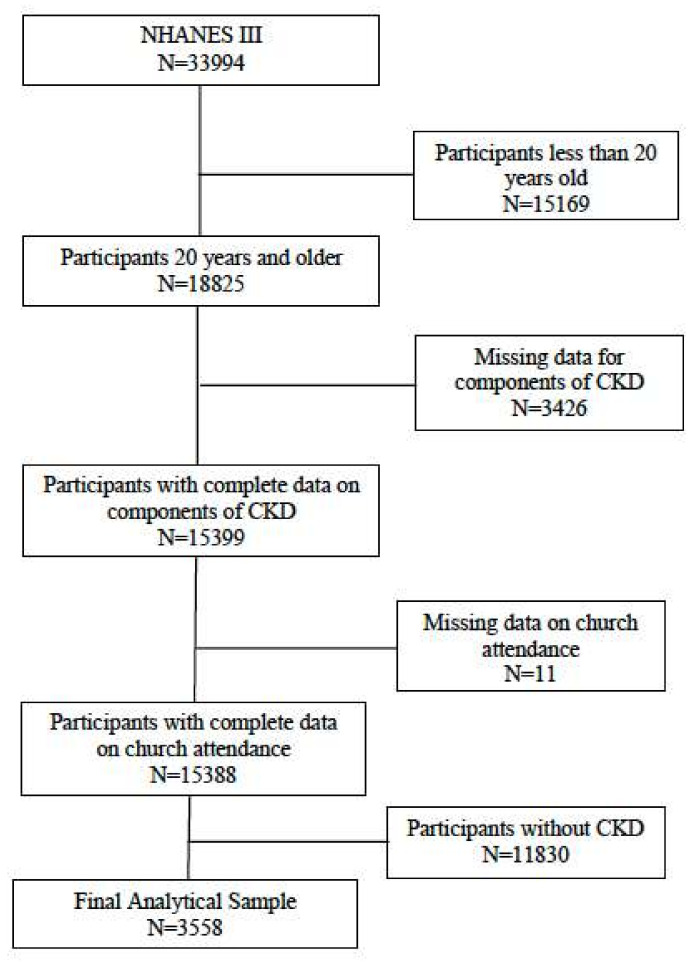
Study Cohort Flow Chart.

The analytic sample was derived from adult participants who were 20 years of age or older at the time of their interview and had CKD. CKD status was defined by single values of estimated glomerular filtration rate (eGFR) using the Chronic Kidney Disease Epidemiology Collaboration (CKD-EPI) equation and/or albumin-to-creatinine ratio [41]. Individuals were defined as having CKD if eGFR <60 mL/min/1.73 m^2^ (not on dialysis), or eGFR ≥ 60 mL/min/1.73 m^2^ and/or albumin-to-creatinine ratio ≥ 17 mg/g for males or ≥ 25 for females [42]. Participants who did not have CKD (*n* = 11,830), were missing data for components of CKD (*n* = 3426), or did not report religious service attendance (*n* = 11) were excluded. The sample size for our analysis was 3558 (Figure 3). Institutional review board approval and informed consent were not required because NHANES III data are publicly available and anonymized. The analysis of these data is not considered human subject research.

### 2.2. Study Variables

The outcome variable for this study was all-cause mortality and was created by the National Center for Health Statistics. The NHANES III Linked Mortality File was used to estimate race-specific related death rates for NHANES III sample members. These mortality data were based on a predictive algorithm linking NHANES III to the National Death Index through 31 December 2015 [43]. The results of this match provided up to 27 years of follow-up (mean [SE] 16 [0.2] years).

The primary independent variable of interest was religious service attendance. This categorical variable was derived from a questionnaire item asking participants “How often do you attend church or religious services? (per year)”. Responses were transformed into 3 categories—“no religious service attendance”, “less than once per week”, and “one or more times per week” [21,22].

Our analyses included covariates that have been identified in the literature as potential confounding factors. We included demographic, socioeconomic, health, and behavioral variables that have been shown to be related to mortality [21,44]. Sex was categorized as male or female, and marital status as never married, yes, or no. The self-reported race and ethnicity variables were derived from five categories in the NHANES dataset (non-Hispanic White, non-Hispanic Black, Mexican American, Other Hispanic, and Other Race including Multi-Racial). Four indicator variables were created representing non-Hispanic Whites, non-Hispanic Blacks, Hispanic (Mexican American and Other Hispanic combined into a single category), and Other racial/ethnic groups.

Socioeconomic status (SES) covariates included education, health insurance status, and income. Education was a 3-category variable indicating whether a participant completed less than 9, between 9 and 12, and more than 12 years of education. Health insurance status was an indicator variable denoting whether a participant self-reported having health insurance. Income was self-reported and defined by an income-to-needs variable (poverty-income ratio), derived from the ratio of household income to the US poverty threshold based on each respondent’s family size and composition at the time of the NHANES III examination [21,44].

The health condition covariates were variables indicating whether participants reported having hypertension, diabetes mellitus, congestive heart failure (CHF), asthma, chronic obstructive pulmonary disease (COPD), non-skin cancer, thyroid disease, and rheumatoid arthritis. Allostatic load was also included in the analysis and was calculated as a summative measure derived from values for clinical/biological markers available in NHANES III that have previously been reported to represent physiological dysregulation [44,45]. These included: cardiovascular (systolic blood pressure, diastolic blood pressure, total cholesterol/high density lipoprotein (HDL) ratio, and heart rate); nutritional/inflammatory markers (albumin and C-reactive protein); and metabolic (waist-hip ratio, BMI, and glycated hemoglobin).

The behavior covariates in our study included physical activity (any vs. none), smoking status (current smoker, former smoker, and never smoker), and alcohol use (never drinks, ≤1 drink/day, and >1 drink per day) [21,44]. Three social support covariates were derived from three survey items: (a) “In a typical week, how many times do you talk on the telephone with family, friends, or neighbors?”; (b) “How often do you get together with friends or relatives; I mean things like going out together or visiting in each other’s homes? (per year)”; and (c) “About how often do you visit with any of your other neighbors, either in their homes or in your own? (per year)”.

### 2.3. Statistical Analyses

Descriptive statistics were used to characterize study participants. Weighted frequencies and means summarized the participant characteristics by religious service attendance. Cox proportional hazards were estimated to produce hazard ratios (HR) and 95% confidence intervals (CI) to determine the association between religious service attendance and mortality among participants with CKD. We used a series of models to examine this association. Model 1 was adjusted for demographic (age, race, and gender) and SES variables. Model 2 added to Model 1 health factors including eGFR, urine albumin to creatinine ratio (UACR) and allostatic load, as well as health conditions not included in the allostatic load. The fully adjusted model added health behaviors and social support covariates to Model 2. Adjusted Kaplan–Meier survival curves were estimated and compared with a log-rank test.

All analyses accounted for the complex sampling design on NHANES III [46]. Final analyses were conducted using SAS software V.9.4 (SAS Institute, Cary, NC, USA), SUDAAN software Release 11.0.3 (SUDAAN Statistical Software Center, Research Triangle Park, NC, USA), and STATA 16 (StataCorp, College Station, TX, USA). *p*-values less than 0.05 were considered statistically significant.

## 3. Results

The sample characteristics presented in Table 1 indicate that 43.5% of participants attended religious services at least once per week while more than one third of the respondents (33.8%) reported that they did not attend religious services. The mean age of the cohort was 58 ± 0.7 years and the average age of the “less than once per week” group was about a decade younger than the other groups. There were statistically significant distinctions in the proportions of racial and ethnic groups across the religious service attendance categories. For example, the largest segment of Non-Hispanic White respondents was that of those who did not attend religious services at all while the largest proportions of the African Americans, Hispanic, and Other Race groups were in the “less than once per week” category. Women were more likely to attend religious services than men. While the prevalence of comorbid conditions varied among the three groups, no one category of religious service attendance consistently had a higher burden of comorbidities than the others. Other covariates had significant statistical differences across religious service attendance categories. Those reporting no religious service attendance were more likely to smoke and to be physically inactive. They also had the greatest number of visits with neighbors (77 ± 1.1), followed by those attending religious services one or more times per week (65 ± 1.1) and three or fewer times per month (55 ± 1.1) (*p* < 0.04). However, there was no statistically significant difference among the number of phone calls or number of home visits with friends or relative among the three groups.

### 3.1. Allostatic Load

The distribution of allostatic load by religious service attendance is shown in Table 2. Participants who did not attend religious services had significantly higher rates of three measures of allostatic load (systolic blood pressure, total cholesterol/HDL ratio, and heart rate) than attendees of religious services. However, there was no significant difference in the overall allostatic load score across religious service attendance categories (*p* = 0.07).

### 3.2. All-Cause Mortality

**Figure 4:**
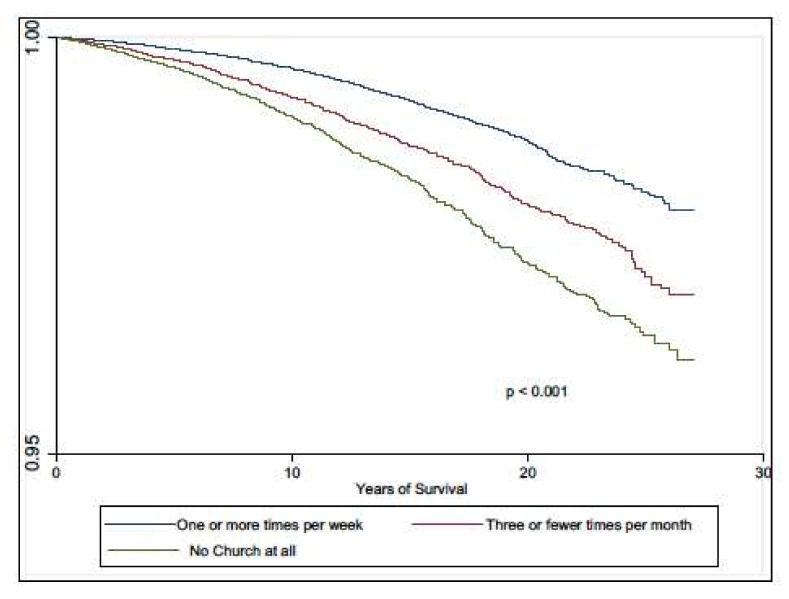
Age-adjusted CKD survival curves.

The association between all-cause mortality and religious service attendance among participants with CKD is shown in Table 3. In the unadjusted model, the mortality risks for participants who attended religious services one or more times per week as well as those who attended less than once per week were lower than their peers with CKD who did not attend religious services. In the fully adjusted model, the hazard ratio coefficient for those who attended religious services less than once per week was no longer significant (HR 0.90; 95% CI 0.73–1.10). However, the mortality risk for participants who attended religious services at least once per week was 21% lower than their peers with CKD who did not attend religious services at all, even after controlling for demographic variables, SES, clinical conditions (such as eGFR, UACR, and allostatic load), health behaviors (including smoking and alcohol consumption), and measures of social support (HR 0.79; 95% CI: 0.64–0.98). The adjusted Kaplan–Meier survival curves presented in Figure 4 indicate that CKD survival was highest in individuals who attended religious services at least weekly and worse in those who did not attend religious services at all.

## 4. Discussion

The objective of our study was to examine the association between religious service attendance (a common measure of religiosity) and mortality risk in a subset of U.S. adults in the general population who have CKD, but are not yet on dialysis. We found that the mortality risk for patients with CKD who attended religious services at least once a week was lower than their peers who did not attend religious services, and those who attended less than once a week had a trend toward a lower risk of death that did not reach statistical significance in the fully adjusted models. This relation appears to be robust as the hazard ratio coefficients were minimally impacted by adjustment for social, demographic, and clinical covariates, including allostatic load, eGFR, and UACR. Although there were differences in the prevalence of religious service attendance by race and ethnicity, we did not find an interaction between them and mortality, suggesting any potential impact of religious service attendance was similar across groups in the sample. These findings emphasize the importance of faith-related variables in biomedical research. In addition, this work makes a significant contribution to the understanding of the factors that may influence mortality among CKD patients.

Our findings are consistent with earlier studies that demonstrate that religious service attendance is associated with increased longevity in the general population [21,22,23]. This is important because traditional cardiovascular risk factors have been found to have paradoxical associations with mortality in patients with CKD [35,36,37] and it was not implicit that religious service attendance would have a similar association with mortality and CKD as in the general population. The association of religious service attendance and mortality in our study is likely multifactorial. Lucchetti and colleagues surveyed 205 dialysis patients in Brazil and found that religiousness was associated with less depressive symptoms and better quality of life [12], while in 63 Thai patients with CKD, spirituality was inversely correlated with depressive symptoms, suggesting that a better understanding of spirituality could lead to the better management of depression and improving survival for patients with CKD [13]. Another possible contributor is the increased social support provided by regular religious service attendance [21,28]. Religious organizations have been settings where individuals can receive guidance and emotional support to help them to cope with challenging life circumstances [28,47,48,49]. Spinale and colleagues found that a high level of self-reported spirituality and social support were associated with increased survival in 166 dialysis patients followed for 19 months, but only social support remained independently associated with mortality when they were controlled for each other suggesting the effect of spirituality in their study was mediated through social support [15]. Spirituality could influence dependence on external coping factors, such as smoking, alcohol consumption, or other behaviors. However, when we controlled the time spent seeking social support from significant others (i.e., family, friends, relatives, and neighbors) as well as health behaviors, we found that the inverse relation between religious service attendance and mortality among individuals with CKD persisted, suggesting that the benefits of attending religious services can extend beyond the social support provided by close friends and family.

Religious service attendance may also be a mechanism to help patients to cope, independent of social support, with the uncertainty, vulnerability, hopelessness, fear, anger, and particularly depression that can accompany chronic conditions [50]. Depression is common among kidney disease patients [5] and has been linked to acute kidney injury [51], progression of kidney disease [52], worse cardiovascular outcomes [53], and increased mortality [7]. Studies have documented that progressive CKD without the hope of transplantation begets the looming prospect of and experience with dialysis that can have an adverse impact on mental, emotional, and familial health [50]. We could not investigate the relation between religious service attendance, depression, and CKD in this study because assessment data were not ascertained in persons over 40 years of age. However, Hill and colleagues reported religiosity was indirectly associated with increased leukocyte telomere length, a biologic marker of longevity, in a large probability sample of 1252 adults aged 22 to 69 in Tennessee [54], likely through reducing symptoms of depression [55]. Thus, assessing depression would be an important next step in assessing the potential mechanisms through which religious service attendance is associated with longevity among CKD patients.

Churches and other places of worship are multifaceted social institutions that can enhance survival through other causal pathways, such as lifestyle decisions, psycho-social influences, and social networks [56,57,58]. For example, Seventh-day Adventists promote and adhere to a predominantly vegetarian diet and this practice can lower risks for diabetes mellitus and hypertension, the two most common causes of CKD [59].

Religious institutions can also be contexts promoting practices linked to holy virtues that have been associated with stress reduction and enhanced resiliency [21]. Churches, mosques, and temples have been known to offer affirmative spiritual messages and activities that help attendees to cope with structural and individual-level stressors [24]. Holiness experienced through the giving and receiving of generosity, hospitality, objectivity, sensitivity, and tolerance can be particularly salient for individuals with CKD as it cultivates inter-connectedness with others and the surrounding environment [60,61] as well as instills meaning, purpose, mindfulness, connection, and wholeness regardless of life circumstances [21,62,63,64,65]. Consistent and frequent engagement with communities with these characteristics can possibly explain enhanced longevity among individuals with CKD attributed to religiosity. Interestingly, Davison and Jhangri examined the religious and existential dimensions of spirituality on HRQOL in patients with CKD on dialysis and found the existential domain of spirituality was more clinically relevant to dialysis patients and had a greater impact on HRQOL compared with measures of religiosity [66]. They did not examine associations with clinical outcomes.

Our study makes a conceptual and empirical contribution to the existing body of research examining religiosity and health. However, it does have some noteworthy limitations. Our religiosity measure of religious service attendance is only one of many practices associated with religious organizations or beliefs. Attending religious services is an outward expression of religiosity; however, our study does not account for personal practices, such as prayer or sacred text reading, and we did not have data on religious affiliation or duration/type of religious activity. As such, it is possible that the findings from this study are conservative. It is also noteworthy that the study period for NHANES III preceded the steady decline in religious decline attendance. Our ability to extrapolate findings for the current day is tempered given changes in the religious landscape. Data used for this research are observational, therefore one cannot infer causality. Although the NHANES enrolls a random representative non-institutionalized sample of the US population, study participants may differ from those who are not participants in subtle ways that may affect both the study results and the generalizability of the findings. In addition, CKD status was defined by single lab values. We adjusted for numerous potential confounders, such as other major health conditions and allostatic load, but we could not exclude residual confounding from items such as risk factor severity/duration, depression, or resilience factors, including compassion and holiness, which may or may not be related to religious service attendance and were not available to be included in the model. Finally, our cohort is likely dominated by people following Judeo-Christian practices and unlikely to capture the diversity and nuances of religion and spirituality in non-Western countries that may manifest differently.

## 5. Conclusions

Our analyses provide evidence of a fairly robust association between religious service attendance and mortality in NHANES III adult participants with CKD despite their limitations. The relation was consistent even after adjusting for demographic, social, behavioral, and clinical factors, including eGFR, UACR, and allostatic load. Our results underscore the potential influence that religious practices or the close association with a religious body can have on health and longevity among individuals with burdensome health conditions, such as CKD. Future studies prospectively examining the effect of religious and/or spiritual activities can yield findings that can contribute to the effort to address the global burden of CKD and its complications.

## Figures and Tables

**Table 1:** Distribution of Sample Characteristics for NHANES III Participants with Chronic Kidney Disease for the Total Sample and by Religious Service Attendance.

	Total (*n* = 3558)	One or More Times per Week (*n* = 1548)	Less Than Once per Week (*n* = 809)	No Religious Service Attendance (*n* = 1201)	*p*-Value
Demographics					
Age (Mean (SE))	58 (0.7)	61 (0.7)	51 (1.2)	59 (1.1)	<0.001
Race/Ethnicity (%)					
White Black Hispanic Other	77.2 12.5 3.9 6.4	80.0 11.2 3.6 5.2	72.2 17.2 4.1 6.5	84.4 8.2 2.0 5.4	<0.001
Sex (%)					<0.001
Male Female	46.0 54.0	41.0 59.0	43.9 56.1	53.4 46.6	
Never Married	9.3	7.7	10.1	10.7	0.562
Socio-Economic Factors					
Education (%)					0.004
<9 years 9–12 years >12 years	21.6 47.4 31.0	20.7 45.5 33.8	19.1 44.0 36.9	24.4 51.8 23.7	
Poor (poverty-income ratio < 2) (%)	41.0	39.2	40.4	43.6	0.292
No health insurance (%)	8.0	6.1	10.2	8.7	0.037
Major Cardio-Renal Comorbid Conditions					
Hypertension (%)	53.1	56.9	44.0	54.7	0.004
Diabetes (%)	21.2	19.8	22.0	22.4	0.515
Congestive Heart Failure (%)	6.8	6.4	6.7	7.2	0.754
eGFR (CKD-EPI) ml/min/1.73 m^2^ (Mean (SE))	81.5 (1.5)	76.6 (1.6)	90.8 (3.2)	81.3 (2.4)	<0.001
UACR (mg/g) (Mean (SE))	126.3 (8.3)	99.6 (9.8)	138.3 (16.8)	150.7 (15.5)	0.005
Mean (SE) allostatic load score (range 0–9) ^a^	2.9 (0.1)	2.8 (0.1)	2.7 (0.1)	3.0 (0.1)	0.07
Comorbid conditions (non-ckd related)					
Lung disease (%)	12.5	13.2	8.9	14.1	0.024
Cancer (%)	7.2	7.0	7.4	7.4	0.943
Thyroid disease (%)	8.4	10.8	5.8	7.3	0.022
Rheumatoid arthritis (%)	7.0	6.0	6.8	8.2	0.131
Asthma (%)	8.3	7.7	9.6	8.0	0.433
Health Behaviors					
Tobacco Use					<0.001
Never smokers (%) Former smokers (%) Current smokers (%)	42.7 34.7 22.6	49.0 36.8 14.2	43.8 30.2 26.0	34.7 35.1 30.1	
Physically active (%)	70.8	72.5	75.0	66.0	0.002
Alcohol Use					0.056
Non-drinkers (%) ≤1 alcoholic drink/day (%) >1 alcoholic drink/day (%)	56.1 36.9 7.0	62.1 31.6 6.3	50.5 43.6 5.9	52.9 38.6 8.5	
Social Support Measures (Mean (Se))					
Number of phone calls with family/friends/neighbors per week.	7.9 (1.0)	7.7 (1.1)	8.6 (1.1)	7.5 (1.1)	0.37
Number of home visits with friends or relatives per year.	72 (1.0)	75 (1.1)	81 (1.1)	65 (1.1)	0.058
Number of home visits with neighbors per year.	66 (1.1)	65 (1.1)	55 (1.1)	77 (1.1)	0.039

Note: Percentages in this table were derived by using NHANES III weighting and design factors to account for its complex sampling design. SE: standard error. CKD-EPI—Chronic Kidney Disease Epidemiology Collaboration equation; eGFR—estimated glomerular filtration rate; UACR—urinary albumin to creatinine ratio. ^a^ The allostatic load score is derived by assigning those with high-risk values on each component a score of “1”. These binary indicators are summed to generate a score ranging from 0 to 9 with higher values indicating higher physiological strain.

**Table 2:** Distribution of Allostatic Load Components of NHANES III Participants with Chronic Kidney Disease for the Total Sample and by Religious Service Attendance.

	Total (*n* = 3558)	One or More Times per Week (*n* = 1548)	Less Than Once per Week (*n* = 809)	No Religious Service Attendance (*n* = 1201)	*p*-Value
Allostatic Load Components (% of each subgroup with “high risk” values) ^a^					
Systolic blood pressure (mmHg)	1563 (37.2)	715 (39.6)	310 (28.0)	538 (40.5)	0.002
Diastolic blood pressure (mmHg)	381 (10.3)	155 (9.4)	109 (10.0)	117 (11.5)	0.58
Waist/hip ratio	2667 (79.9)	1206 (80.6)	608 (75.0)	853 (82.4)	0.08
Total cholesterol/HDL ratio	1307 (39.6)	598 (38.5)	267 (36.9)	442 (42.7)	0.04
Glycated hemoglobin (%)	1589 (37.0)	722 (35.9)	346 (36.5)	521 (38.7)	0.64
Heart Rate (beats/min)	150 (6.0)	59 (4.1)	29 (6.7)	62 (8.1)	0.01
Albumin (g/dL)	689 (16.2)	280 (15.2)	163 (15.6)	246 (17.8)	0.35
C-reactive protein (mg/L)	1633 (42.8)	681 (40.5)	364 (44.2)	588 (44.6)	0.32
Body Mass Index (kg/m^2^)	987 (28.9)	412 (26.6)	248 (30.1)	327 (30.9)	0.33
Mean (SE) allostatic load score (range 0–9) ^b^	2.9 (0.1)	2.8 (0.1)	2.7 (0.1)	3.0 (0.1)	0.07

^a^ High-risk values were based on clinical cut points that include systolic blood pressure > 140 mmHg; Diastolic blood pressure > 90 mmHg; waist/hip ratio > 0·9 (Males) & waist/hip ratio > 0.85 (Females); Chol/HDL > 5; HbA1c > 5.7; Heart rate > 90; albumin <3.8; C-reactive protein ≥ 0.3; Body mass index >30; SE: standard error. ^b^ The allostatic load score is derived by assigning those with high-risk values on each component a score of “1”. These binary indicators are summed to generate a score ranging from 0 to 9 with higher values indicating higher physiological strain.

**Table 3:** Hazard ratios and 95% Confidence Intervals for the Association between All-Cause Mortality and Religious Service Attendance among NHANES III Participants with CKD.

	Unadjusted	Model 1	Model 2	Model 3
No religious service attendance (*n* = 1201)	Reference	Reference	Reference	Reference
Less than once per week (*n* = 809)	0.59 (0.49–0.70)	0.84 (0.72–0.99)	0.90 (0.76–1.06)	0.90 (0.73–1.10)
One or more times per week (*n* = 1548)	0.88 (0.78–0.99)	0.72 (0.64–0.81)	0.82 (0.72–0.92)	0.79 (0.64–0.98)

Model 1 adjusts for age, race, sex, and socioeconomic status. Model 2 adds comorbidities, estimated glomerular filtration rate, urinary-albumin-to-creatinine ratio, and allostatic load to the covariates in Model 1. Model 3 adds health behavior and social support variables to the covariates in Model 2.

## Data Availability

All of the data used in the study are publicly available from the Centers for Disease Control and Prevention.
